# Dataset on non-carcinogenic risk via nitrate and nitrite in the groundwater of Divandarreh County, Kurdistan province, Iran: A potential concern for drinking

**DOI:** 10.1016/j.dib.2018.09.035

**Published:** 2018-09-15

**Authors:** Abotaleb bay, Shakir Ali, Mansoureh Ghezelsofla, Hassan Keramati, Bigard Moradi, Yadolah Fakhri

**Affiliations:** a Environmental Health Research Center, Golestan University of Medical Sciences, Golestan, Iran; b Department of Geology, University of Delhi, 110007, India; c Master of Science in Environmental Science, Faculty of Science, Shahid Rajaee Teacher Training University, Tehran, Iran; d Social Determinants of Health Research Center, Semnan University of Medical Sciences, Semnan, Iran; e Research Center for Environmental Determinants of Health (RCEDH), Kermanshah University of Medical Sciences, Kermanshah, Iran; f Department of Environmental Health Engineering, School of Public Health, Student Research Committee, Shahid Beheshti University of Medical Sciences, Tehran, Iran

**Keywords:** Nitrate, Nitrite, Groundwater contamination, Risk assessment, Rural Iran, Divandarreh County

## Abstract

The presence of elevated nitrate (NO_3-_) and nitrite (NO_2-_) concentration in drinking water higher than the standard limits could endanger the health of consumers. For this data article, concentration of NO_3-_ and NO_2-_ was measured in 118 samples collected from 59 active rural wells in Divandarreh County and the non-carcinogenic risk in the adults and children was estimated by Monte Carlo simulation (MCS). The obtained data showed that the average concentration of NO_3-_ and NO_2-_ was ranges from 31.37 ± 18.87 mg/L and 1.45 ± 0.90 mg/L respectively. Based on acquired data, NO_3-_ concentrations were 37 times higher than NO_2-_ with significant p value of < 0.05. The average concentration of NO_3-_ and NO_2-_ was lower than the national standard with p value < 0.05. However, the concentration of NO_3-_ and NO_2-_ in 23.7% and 13.5% of wells was higher than the national standard of Iran. Total target hazard quotient (TTHQ) in the adults and children was 1.78 and 1.54, respectively. Although, the average concentration of NO_3-_ and NO_2-_ in drinking water was lower than the national standard limits, but the non-carcinogenic risk assessment showed that the children and adults are at a significant risk via nitrate and nitrite in the rural Divandarreh County (TTHQ > 1).

**Specifications table**

**Table:** 

Subject area	Environmental sciences
More specific subject area	Environmental chemistry
Type of data	Table and figure
How data was acquired	For this data article, concentration of NO_3-_ and NO_2-_ was measured in 118 samples collected from 59 active rural wells in Divandarreh County and the non-carcinogenic risk in the adults and children was estimated by Monte Carlo simulation (MCS).
Data format	Raw, analyzed
Experimental factors	The wavelength for determination of nitrate and nitrite by emission spectroscopy method are 500 and 507 nm, respectively.
Experimental features	The samples collection and nitrate and nitrite ions analysis was performed according to the standard method.
Data source location	Divandarreh county, Kurdistan province, Iran
Data accessibility	Data are included in this article
Related research article	X. Su, H.Wang, Y .Zhang, Health risk assessment of nitrate contamination in groundwater: a case study of an agricultural area in Northeast China, Water. Resourc. Manage. 27(2013)3025–34 [1].

**Value of the data**

● Nitrate and nitrite are one of the most common contaminants in drinking water in Iran [2], [3], [4], [5]. Therefore, monitoring these two pollutants and assessing their associated health risks (provided in this data article) will be very beneficial for the selection of safe drinking water sources.
● The obtained data can provide useful information on the quality of drinking groundwater (wells) in the Divandarreh County, in terms of nitrite and nitrate.
● The acquired data can be useful for management plans for drinking water.
● The effect of nitrate and nitrite on human health is assessed via Monte Carlo simulation (MCS) method. Therefore, this evaluation method in present data article can be useful and applicable for future similar studies.

## 1 Data

### 1.1 Concentration of nitrate (NO_3-_) and nitrite (NO_2-_)

Concentration of NO_3-_ and NO_2-_ was measured in 118 samples collected from 59 active rural wells. The minimum and maximum concentration of NO_3-_ in both spring and autumn seasons was observed in Tazeh Abad Ghaziali (0.9 mg/L) and Gorbabaali (134 mg/L) rural localities (Table 1). While, the minimum level of NO_2-_ in the spring season was reported from Zaki Bigalia (ND), Hazarkanian (ND) and Gorbabaali (ND) and maximum concentration was observed in Vazman rural (5.6 mg/L) locality (Table 1).

**Table 1:** Concentration of Nitrate and Nitrite in 59 rural localities of Divandarreh County, Iran.

**Rural name**	**Latitude**	**Longitude**	**Nitrate (mg/L)**			**Nitrite (mg/L)**		
			**Spring**	**Autumn**	**Average**	**Spring**	**Autumn**	**Average**
Dar asb	697,952	3,987,484	48.00	42.00	45.00	0.01	0.02	0.02
Bash ghshlagh	683,947	3,999,916	25.00	18.00	21.50	0.02	0.02	0.02
Darband	700,662	3,977,609	20.20	13.20	16.70	0.02	0.04	0.03
Dalan	699,013	3,973,730	16.00	12.10	14.05	0.03	0.03	0.03
Kalhor abad	688,215	3,947,622	29.00	19.00	24.00	0.03	0.05	0.04
Bayz yadabad	668,997	4,016,853	22.00	18.00	20.00	0.05	0.04	0.04
Ghleh reyhaneh	674,959	3,975,291	25.00	11.60	18.30	0.05	0.05	0.05
Ahmad kar	670,886	3,995,782	32.10	23.80	27.95	0.04	0.06	0.05
Shja abbad	674,470	3,963,890	30.00	24.00	27.00	0.04	0.06	0.05
Ziki big alai	673,237	4,008,669	29.00	24.10	26.55	ND	0.05	0.05
Sarab gherh khan	711,010	3,997,938	31.90	23.00	27.45	0.05	0.06	0.05
Ebrahim abbad	665,115	3,982,711	35.50	34.40	34.95	0.05	0.06	0.05
Kani shirin	681,921	4,013,914	40.40	34.40	37.40	0.05	0.06	0.06
Ghleh kohneh	696,522	4,002,364	48.00	45.00	46.50	0.05	0.07	0.06
Katak	674,447	3,981,147	37.20	31.00	34.10	0.06	0.06	0.06
Kani shirn	682,341	4,014,723	64.20	17.50	40.85	0.04	0.08	0.06
Kani chayi	679,868	3,997,723	49.40	46.00	47.70	0.06	0.07	0.07
Goomehi	666,685	4,002,175	37.00	35.50	36.25	0.06	0.08	0.07
Gadmeh getter	708,022	3,995,250	57.00	51.00	54.00	0.07	0.07	0.07
Heydar dideh ban	686,465	4,004,294	60.00	53.00	56.50	0.07	0.08	0.07
Ghaleh rootelh	681,598	3,990,604	57.00	74.00	65.50	0.08	0.07	0.08
Shaali shel	683,994	4,005,054	6.00	43.00	24.50	0.07	0.10	0.09
Radhid abbad	684,050	3,981,000	40.00	14.30	27.15	0.09	0.09	0.09
Bardeh resheh	667,004	4,007,168	24.10	22.00	23.05	0.09	0.10	0.10
Kalkan	673,339	3,987921	40.00	25.00	32.50	0.10	0.10	0.10
Seyer ali	676,654	4,002,938	36.00	32.00	34.00	0.06	0.15	0.11
Tazeh abbad vazir	701,701	3,985,774	29.00	18.50	23.75	0.12	0.14	0.13
Khaki big	672,222	4,005,422	42.00	33.00	37.50	0.10	0.18	0.14
Youz bashi kenedi	670,320	4,019,397	18.00	16.00	17.00	0.14	0.16	0.15
Ali abbad kerfeto	663,074	4,015,530	50.00	34.40	42.20	0.12	0.20	0.16
Jiran mango	667,248	3,997,466	38.40	33.10	35.75	0.03	0.30	0.17
Ghebi soor	687,867	3,967,563	35.00	25.42	30.21	0.12	0.22	0.17
Seyr sofla	679,204	4,002,392	31.00	24.00	27.50	0.19	0.20	0.20
Alijan	651,667	3,976,515	51.00	45.00	48.00	0.10	0.34	0.22
Tazeh abbad maran	682,562	4,016,289	38.00	30.80	34.40	0.30	0.30	0.30
Maran alia	677,106	4,010,170	30.14	19.00	24.57	0.10	0.80	0.45
Abb barik	702,412	3,998,803	40.00	38.00	39.00	0.09	1.00	0.55
Zafar abbad	678,180	3,988,511	29.10	27.00	28.05	0.60	0.60	0.60
Ghar agol	699,875	4,000,211	36.30	25.10	30.70	0.18	1.40	0.79
Zaki big sofla	675,670	4,006,131	28.00	19.80	23.90	0.81	0.84	0.83
Ali abbad maran	673,493	4,015,657	26.00	16.00	21.00	1.60	0.30	0.95
Morad ghloi	711,662	3,992,611	45.00	39.00	42.00	1.00	1.30	1.15
Gol tapeh alia	669,618	3,999,376	21.00	21.30	21.15	1.00	1.40	1.20
Papaleh	694,851	4,003,853	68.80	48.80	58.80	1.20	1.50	1.35
Kas nzan	677,961	3,994,592	26.00	19.00	22.50	1.20	1.60	1.40
Ghjan	680,603	4,007,129	21.00	26.90	23.95	0.10	3.00	1.55
Hossen abbad maran	671,410	4,012,546	123.00	63.50	93.25	1.40	2.00	1.70
Sharif abbad	659,098	3,977,126	36.00	23.00	29.50	1.90	2.40	2.15
Gorr baba ali	667,920	4,011,063	134.00	77.00	105.50	5.00	0.80	2.90
Darvishan	658,642	3,953,591	26.00	23.00	24.50	3.00	3.20	3.10
Sar ghaleh	691,282	3,978,327	28.60	19.50	24.05	3.00	3.60	3.30
Aghbelagh	692,336	3,953,816	8.40	8.50	8.45	3.00	3.80	3.40
Kos anbar	659,053	3,946,900	4.00	3.00	3.50	4.30	4.50	4.40
Tarz abbad ghazi ali	655,032	3,942,337	0.90	1.30	1.10	5.00	4.40	4.70
Darvyan farsi	650,015	3,948,737	5.00	3.00	4.00	5.00	5.30	5.15
Vazman	644,630	3,978,902	5.40	4.80	5.10	5.60	6.00	5.80
Hezar kanian	663,786	3,959,608	18.10	15.60	16.85	ND^a^	ND	ND
Tazeh abbad baharestan	694,606	3,946,783	10.00	6.25	8.13	ND	ND	ND

a Not detected

The average concentration of NO_3-_ in the 23.7% groundwater samples (14 localities) was found to be higher than the national standard limit (50 mg/L). The average concentration of NO_2_^-^ in 13.5% samples (8 localities) was also higher than the national standard limit. The average concentration of NO_3_^-^ (31.37±18.87 mg/L) and NO_2_^-^ (1.45 ± 0.9 mg/L) was lower than the national and WHO standard limit, significantly (p value < 0.05) (Table 2).

**Table 2:** Concentration of nitrate and nitrite in rural of Divandarreh County, Iran.

**Contaminants**	**Range (mg/L)**	**Median**	**Average ± SD**
Nitrate	1.1–105.5	27.48	31.37 ± 18.87
Nitrite	0.02–5.8	0.15	1.45 ± 0.90

**Fig. 1:**
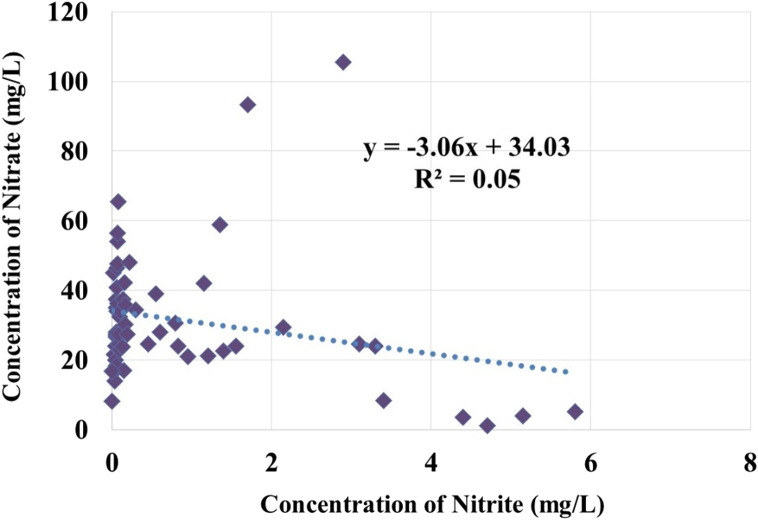
Bivariant plot between nitrate and nitrite in the wells of rural Divandarreh County, Iran.

The results of Pearson correlation analysis showed a non-significant correlation (P value > 0.05) between NO_3-_ and NO_2-_ concentration (Fig. 1). The difference in the biological or chemical reactions could be the probable cause for insignificant correlation between NO_3-_ and NO_2-_ concentration [6], [7]. Similarly, earlier study conducted by Amarlooei et al. in Iran also suggests insignificant correlation between NO_3-_ and NO_2-_ concentration [8].

In Divandarreh County, the concentrations of NO_3-_ was 37 times higher than NO_2-_ concentration, with significant P value of < 0.05 during autumn while, NO_3-_ and NO_2-_ suggests insignificant P value of > 0.05 in summer season.

### 1.2 Health risk assessment

**Fig. 2:**
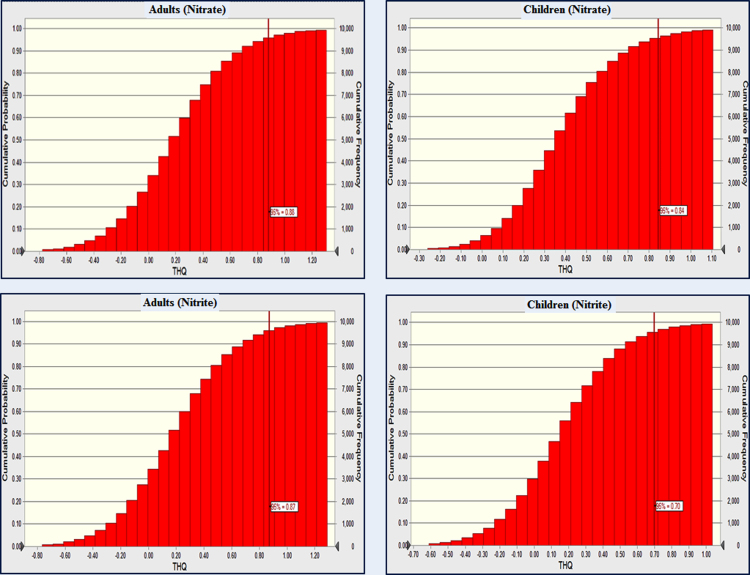
THQ in adults and children due to ingestion of drinking water containing high level of nitrate and nitrite.

THQ in the children and adults due to NO_3-_ was 0.84 and 0.88 and NO_2-_, 0.78 and 0.87 respectively (Fig. 2). THQ in the adults was observed to be 13% higher than those of children. Further, TTHQ in the adults and children was 1.78 and 1.54.

## 2 Experimental design, materials and methods

### 2.1 Study area

**Fig. 3:**
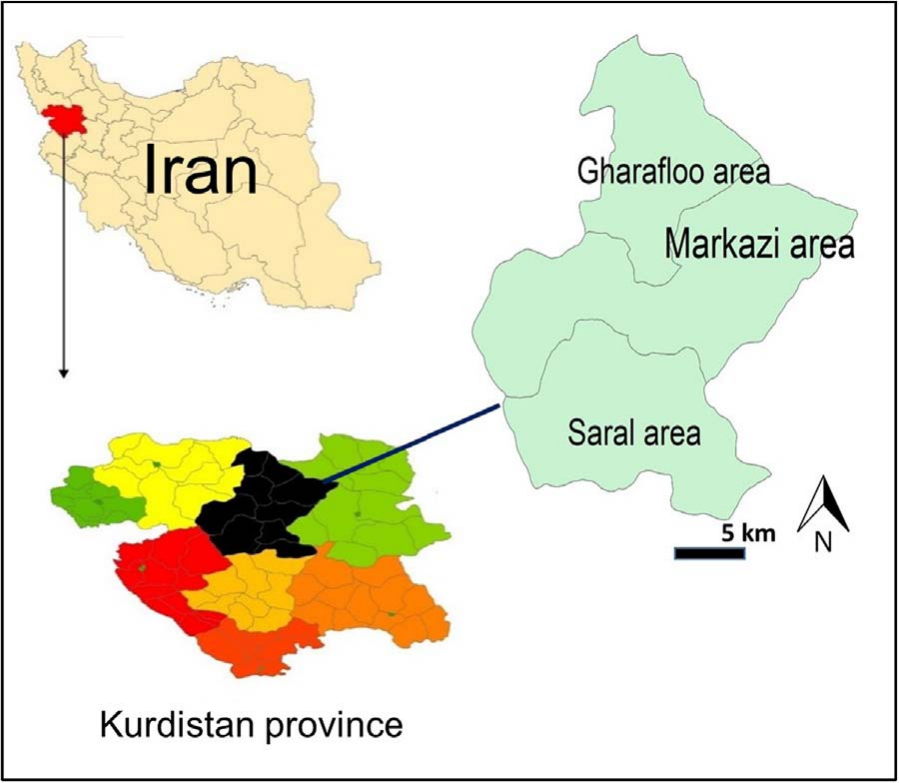
Location of Divandarreh County in Kurdistan province, Iran.

The Divandarreh County (35.9137°N and 47.0267°E) is located at 98 km North of Sanandaj city covering an area of around 4203 km^2^ and at 1850 m above mean sea level (Fig. 3). Divandarreh County experiences cold weather with temperature ranges from 20 to 32 °C throughout the year and receives an average rain of 500 mm/y. According to the latest census of Iran conducted in 2016, the population living in 98 rural areas in Divandarreh County was around 58,503.

### 2.2 Sampling and analysis

A total of 118 samples of groundwater from 59 active wells were collected during spring and autumn of 2016. The collected samples in the glass bottle were transferred to the laboratory of Rural Water and Wastewater Company (RWWC) in Kurdistan Province [9]. Concentration of NO_3_^-^ and NO_2_^-^ in water samples was measured by spectrophotometry UV (HACH DR/5000) in 220 and 507 nm wavelength.

The methods for analyzing the concentration of NO_3-_ and NO_2-_ was cadmium reduction (8039) and diazinon (10207), respectively. Limit of detection (LOD) in the cadmium reduction and diazinon methods was 0.3 mg/L for NO_3-_ and 0.05 mg/L for NO_2-_ [9], [10].

### 2.3 Non-carcinogenic risk

#### 2.3.1 Target Hazard Quotient

The Target Hazard Quotient (THQ) for the exposed population was calculated by the Environmental Protection Agency (EPA) method (Eq. (1)) [11], [12].

(1) $$\mathrm{THQ}=\frac{{C\times E}_{F}\times E_{D}\times W_{\mathrm{IR}}}{\mathrm{RfD}\times {\mathrm{AT}}_{n}}$$

All parameters of Eq. (1) is shown in Table 3.

**Table 3:** Included parameter for estimate THQ in the adults and children.

**Variable**	**Define**	**Unit**	**Value**	**Reference**
C	Concentration	mg/L	–	
E_F_	Exposure frequency	day/year	365	[10]
E_D_	Exposure duration	year	Adults : 70 ; children : 6	[10]
W_IR_	Water ingestion rate	ml/kg-d	Adults : 25 ; children : 20	[12]
AT_n_	Average time	day	Adults : 25,550 ; children : 2190	[12]
RfD	Reference dose	mg/kg-d	Nitrate:1.6 and Nitrite : 0.1	[10]

THQ > 1 suggests the consumer population is at a significant risk of non-carcinogenicity while THQ ≤1 indicates, consumer population are safe w.r.t risk for non-carcinogenicity [12], [13]:

#### 2.3.2 Total target hazard quotient

Total target hazard quotient (TTHQ) in the consumer population due to NO_3_^-^ and NO_2_^-^ was calculated by EPA method (Eq. (2)) [11]:

(2) TTHQ = THQ-NO_3-_ + THQ-NO_2-_

TTHQ value more than 1 shows the consumer population is at a significant risk of non-carcinogenicity, while TTHQ value less than 1, indicates insignificant risk for non-carcinogenicity [14], [15].

### 2.4 Monte Carlo Simulation model

The Monte Carlo Simulation (MCS) is one of the most commonly used models for estimating the probable health risk. In this model, the range of variables as well as other uncertainties are considered for accurate health risk estimation [16], [17]. The worse scenario of health risk of population in the study area was determined based on MCS model (percentile 95% of THQ).

### 2.5 Statistical analysis

Statistical analysis was performed by Kolmogorov-Smirnov test (KS). Since the data were normal distribution (P value > 0.05), for comparison of NO_3-_ and NO_2-_ concentrations with standard values, one sample t test was used. The significant level was P value <0.05.
